# Synchronous adrenal metastasis and an inferior vena cava tumor thrombus from an ovarian carcinoma

**DOI:** 10.1186/1757-2215-7-5

**Published:** 2014-01-13

**Authors:** Hiroyuki Tokue, Azusa Tokue, Yoshito Tsushima

**Affiliations:** 1 Department of Diagnostic and Interventional Radiology, Gunma University Hospital, Gunma, Japan

**Keywords:** Adrenal metastasis, Ovarian carcinoma, Tumor thrombus

## Abstract

A 60-year-old woman presented with synchronous adrenal metastasis and an inferior vena cava tumor thrombus in the adrenal vein that developed from an ovarian carcinoma. The patient underwent total abdominal hysterectomy, bilateral salpingo-oophorectomy, and right adrenalectomy with caval tumor thrombectomy for treatment. Microscopic examination revealed a clear cell ovarian carcinoma and a metastatic adrenal tumor. The patient is clinically free of disease after 6 years of follow-up. There have been no reports of synchronous adrenal metastasis with an inferior vena cava thrombus that developed from an ovarian carcinoma. As several reports have described the long-term survival after adrenalectomy for the treatment of isolated adrenal metastasis, clinicians should be aware of this potential occurrence so that patients can be appropriately treated.

## Background

Adrenal metastases from visceral carcinoma are uncommon; they most commonly arise from primary lung, breast, and kidney tumors [1]. Adrenal metastases from ovarian carcinomas are extremely rare. Currently, there is only one report in the English literature of synchronous adrenal metastasis that developed from an ovarian carcinoma [2], and, to the best of our knowledge, there are no reports of synchronous adrenal metastasis with an inferior vena cava (IVC) thrombus that developed from an ovarian carcinoma. Several investigators have documented that aggressive surgical resection of an adrenal metastasis, when done in patients with solitary, excisable disease and after a long disease-free interval, can prolong patient survival [3]. We present a rare case of synchronous adrenal metastasis with an IVC tumor thrombus in the adrenal vein that developed from an ovarian carcinoma. The patient is clinically free of disease after 6 years of follow-up.

### Case presentation

**Figure 1:**
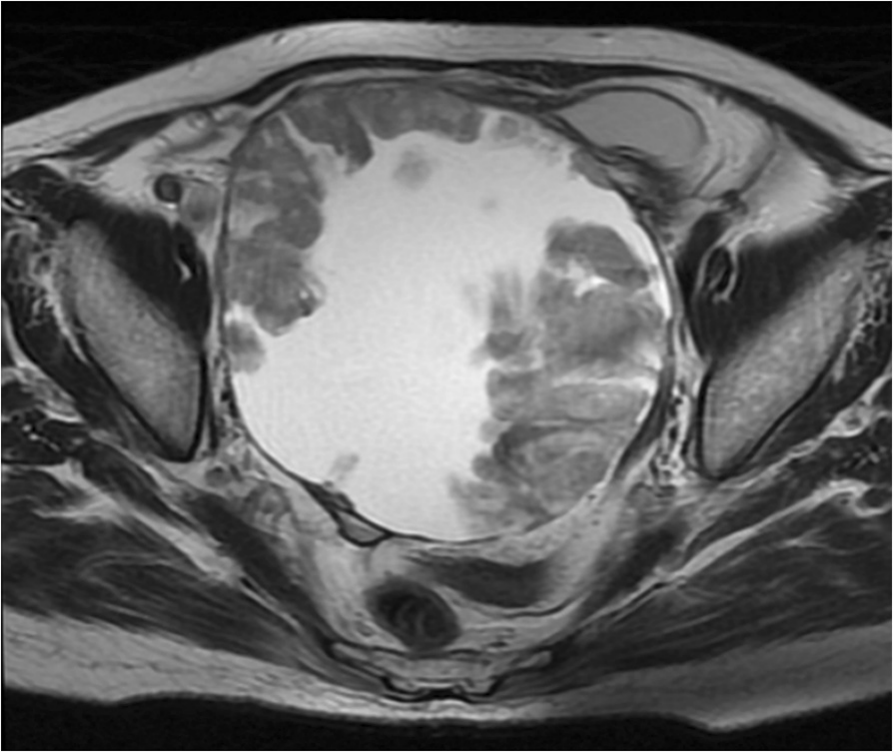
**Pelvic magnetic resonance imaging.** An axial T2-weighted image—of a 60-year-old woman presenting with an ovarian tumor—revealed a cystic mass with solid portions.

**Figure 2:**
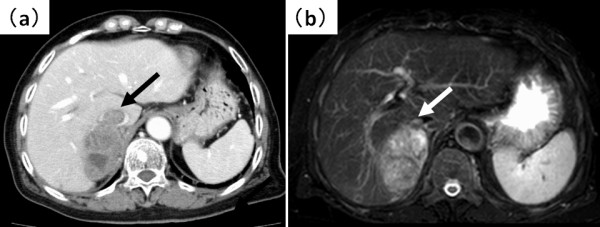
**Abdominal computed tomography(CT)and magnetic resonance imaging. (a)** Contrast enhanced CT and **(b)** fat-suppression T2-weighted images revealed a mass extending through the right adrenal vein into the inferior vena cava (IVC). Arrows designate the IVC tumor thrombus.

**Figure 3:**
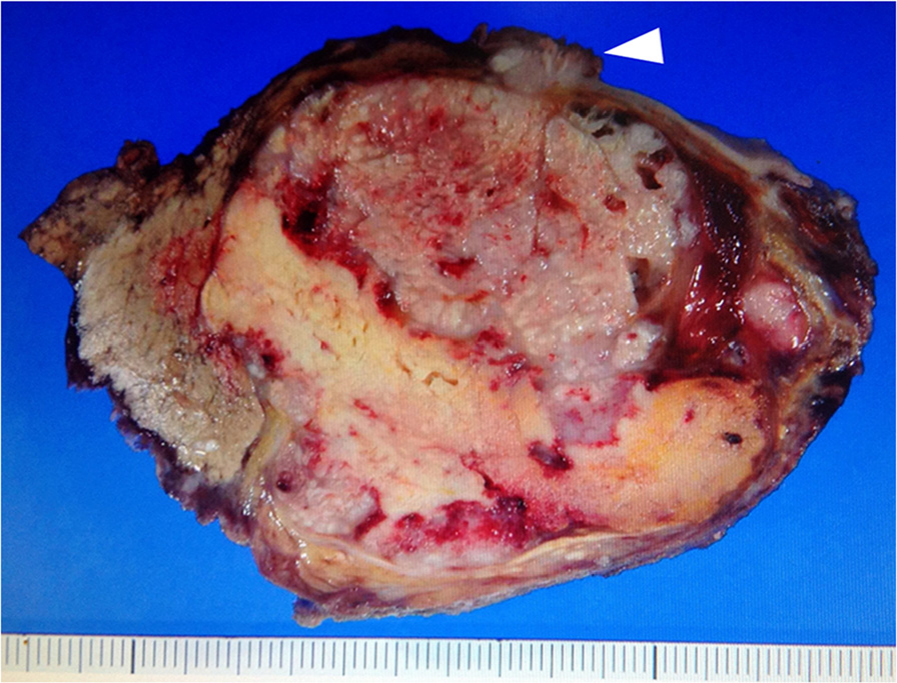
**Macroscopic findings.** Macroscopically, the right adrenal mass had a cream-colored appearance with hemorrhagic areas. The mass extended through the adrenal vein and into the inferior vena cava. Identifiable bright yellow adrenal cortical tissue was notably stretched over the mass. The arrowhead designates the tumor thrombus.

**Figure 4:**
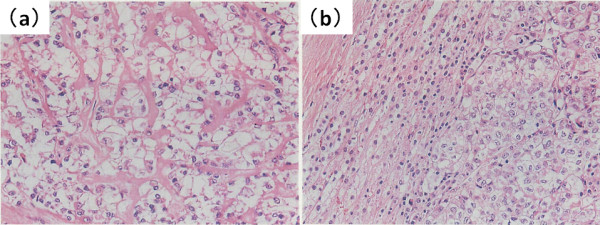
**Histopathological findings. (a)** Histopathological examination (hematoxylin and eosin staining) revealed a clear cell carcinoma in the ovarian tumor (magnification, 200×). **(b)** A clear cell carcinoma similar to that of the primary ovarian carcinoma was confirmed in the adrenal tumor (magnification 200×).

A 60-year-old woman—with no history of tumors—was admitted to the hospital because of abdominal distension. Abdominal ultrasonography and magnetic resonance imaging (MRI) revealed a pelvic mass with solid components and a 20-cm left ovarian tumor, respectively (Figure 1). Computed tomography (CT) and MRI revealed a 9-cm mass extending through the right adrenal vein into the IVC (Figure 2a, 2b). Cervical and endometrial cytology results were normal, and routine laboratory tests were within normal limits. The patient’s serum cancer antigen 125 (CA125) level was 5310 U/mL (normal range: 0–35 U/mL). On the basis of these results, the right adrenal mass was considered a metastasis that developed from the ovarian carcinoma. Although the left ovarian tumor was adhered to the sigmoid colon, laparotomy revealed no dissemination into the abdominal cavity and peritoneal fluid cytology was negative. Total hysterectomy, bilateral salpingo-oophorectomy, low anterior resection, right adrenalectomy with caval tumor thrombectomy, lymphadenectomy, and omentectomy were performed. The tumor thrombus was adhered to the right adrenal vein ostium, and a portion of the IVC wall was resected en bloc by primary adrenal resection. The adrenal gland was carefully mobilized, taking care not to injure the tumor capsule and cause dissemination of the tumor cells. Macroscopic observations revealed that the tumor thrombus, which had extended through the adrenal venous system, was removed intact (Figure 3). Histopathological examination revealed that the left ovarian tumor was a clear cell carcinoma (Figure 4a). Microscopic examination revealed clear cell carcinoma metastases with a tumor thrombus in the right adrenal gland (Figure 4b). There were no metastases in the omentum and paraaortic lymph nodes. The patient’s post-operative course was uneventful, and she was discharged on the tenth operative day. The lymphocele resolved within a week without any sequelae. After the surgery, the patient received chemotherapy with cisplatin and irinotecan. Two years after the surgery, the patient’s CA125 levels were within normal limits (32 U/mL), and she is clinically free of disease after 6 years of follow-up, with no other detectable sites of metastasis.

## Discussion

Ovarian cancer can spread via peritoneal implantation, lymphatic invasion, or hematogenous dissemination. Although intraperitoneal implantation is the primary mode of ovarian cancer dissemination, hematogenous metastases are uncommon. However, autopsy and cross-sectional imaging studies have determined that the prevalence of metastases in advanced disease is higher than the prevalence that was previously recognized. The reported prevalence of adrenal and pancreatic metastases in patients with ovarian cancer at autopsy is 15% and 21%, respectively [4]. Hematogenous metastases from ovarian carcinoma may be more commonly identified with the implementation of new treatments, which would result in improved survival rates.

While the abdominal cavity is routinely surveyed using CT before the treatment of ovarian cancer, incidental discovery of an adrenal mass is rare, even in patients with advanced disease. Table 1 summarizes both the previously reported cases of adrenal metastasis that developed from ovarian cancer and our current case [2]. To the best of our knowledge, there have been no reports of synchronous adrenal metastasis with an inferior vena cava thrombus that developed from an ovarian carcinoma. The adrenal metastasis in the case of our patient was larger than that in previous reports.

**Table 1:** Documented cases of adrenal metastasis that developed from ovarian cancer

**First author**	**Age(years)**	**Location of ovarian tumor**	**Histology**	**Location of adrenal tumor**	**Size(mm)**
Baron [2]	62	Right	Papillary adenocarcinoma	Left	NS
Einat [5]	68	Right	Papillary adenocarcinoma	Left	50
Patlas [6]	57	NS	Serous adenocarcinoma	Left	20
Sundersigh [7]	31	Bilateral	Small cell neuroendocrine	Bilateral	5?×?27?×?28 35?×?33?×?26
Ozapacaci [8]	71	NS	Small cell neuroendocrine	Left	NS
Tokue (present)	60	Left	Clear cell carcinoma	Right	90

An additional 4 cases have been previously reported [9-11]; however, their details are not described. We present the only case of synchronous adrenal metastasis with an inferior vena cava tumor thrombus that developed from ovarian carcinoma.

NS: data not shown.

There are potentially a number of routes for the development of adrenal metastases from an ovarian carcinoma, including the systemic venous, arterial, and lymphatic routes of dissemination. Some reports suggested that the incidence of contralateral lymph node metastasis from the primary site of the ovarian cancer was 11–30% [12,13]. Although the mode of dissemination for contralateral adrenal metastasis is unknown, contralateral lymph node metastasis might suggest a route via the lymphatic system from the primary tumor to the contralateral adrenal gland.

The management of patients with adrenal metastases poses a therapeutic challenge. Although adrenal metastases were considered incurable, surgery is now recommended for isolated adrenal metastasis. Adrenalectomy for metastatic cancer was rarely performed because the survival benefit for patients undergoing such resections was not clear; however, it is now advised in patients with a good performance status where there is unilateral adrenal metastasis with no residual primary or other distant metastases [7,14].

In the case of this patient, the metastases were localized to the adrenal gland and tumor thrombus. Macroscopically complete resection with subsequent chemotherapy contributed to the improved prognosis.

## Conclusions

To the best of our knowledge, we present the first case of synchronous adrenal metastasis with an IVC tumor thrombus through the adrenal vein from an ovarian carcinoma. This is an uncommon case; however, clinicians should be aware of this occurrence so that patients can be treated appropriately. We believe that patients with macroscopically complete tumor resection may benefit from surgical intervention.

### Consent

Written informed consent was obtained from the patient for publication of this Case report and any accompanying images. A copy of the written consent is available for review by the Editor of this journal.

## Competing interests

The authors declare that they have no competing interests.

## Authors’ contributions

HT, AT, YT, All authors read and approved the final manuscript.
